# Acute exacerbation of IPF has systemic consequences with multiple organ injury, with SRA^+^ and TNF-α^+^ cells in the systemic circulation playing central roles in multiple organ injury

**DOI:** 10.1186/s12890-016-0298-x

**Published:** 2016-11-03

**Authors:** Iwao Emura, Hiroyuki Usuda

**Affiliations:** Department of Surgical Pathology, Japanese Red Cross Nagaoka Hospital, 2-297-1 Senshyuu, Nagaoka City, Niigata Prefecture Japan

**Keywords:** Acute exacerbation, Autopsy, Multiple organ injury, Peripheral blood, Scavenger receptor A, Tumor necrosis factor-α

## Abstract

**Background:**

The pathophysiologic mechanisms underlying acute exacerbation of idiopathic pulmonary fibrosis (IPF) are not fully understood. Few studies have examined autopsy findings in patients who have died from an acute exacerbation of IPF. The pathologic findings in systemic organs have not been described.

**Methods:**

We retrospectively reviewed the autopsy findings in 12 patients who had died from an acute exacerbation of IPF and two of connective tissue disease- associated interstitial lung disease between 2005 and 2015. We recorded demographic and clinical characteristics, autopsy findings and cytologic findings in peripheral blood.

**Results:**

The median age at autopsy was 68 years (range 45–87 years); 11 subjects (78.5 %) were men. High-dose corticosteroid, cyclophosphamide and oxygen therapy had been administered to all patients. Underlying lesions had the usual interstitial pneumonia pattern; diffuse alveolar damage and contraction band necrosis were observed in all cases. Large cells expressing scavenger receptor A (SRA^+^) had been observed in the systemic circulation of 11 of the 14 cases (78.6 %) before acute exacerbation, and cells expressing tumor necrosis factor-α (TNF-α^+^) were detected after its diagnosis in nine (64.3 %). Both were detected in all cases at autopsy. There was neutrophil and platelet accumulation predominantly in capillaries, and extensive capillary endothelial cells injury.

**Conclusions:**

Our findings suggest that acute exacerbation of IPF has systemic consequences with multiple organ injury, with SRA^+^ and TNF-α^+^ cells in the systemic circulation playing central roles in multiple organ injury.

## Background

Many patients with idiopathic pulmonary fibrosis (IPF) may experience sudden worsening of respiratory symptoms [1]. This frequently occurs without an identifiable cause, and is termed acute exacerbation of IPF [2]. Acute exacerbations have now also been reported in patients with connective tissue disease (CTD)-associated interstitial lung disease (ILD) and other ILDs [3–5].

The pathophysiologic mechanisms underlying an acute exacerbation are not fully understood [6]. Idiopathic pulmonary fibrosis is defined as a specific form of interstitial pneumonia limited to the lung [7]. At autopsy, histopathologic evidence of diffuse alveolar damage (DAD) is frequently observed in patients who have died from an acute exacerbation of IPF [5, 8, 9]. This finding is shared with the acute respiratory distress syndrome (ARDS) [10] which is considered to be a systemic multi-organ disease [11–13]. Nonetheless, the existing pathologic evidence base in IPF and CTD-associated ILD do not include an assessment of the peripheral blood or systemic organs in patients who have died of an acute exacerbation [5, 8, 9, 14].

We have previously reported that cells expressing scavenger receptor A (SRA) and tumor necrosis factor-α (TNF-α) can be identified at autopsy in the systemic circulation of patients who had died with multiple organ dysfunction syndrome (including those with ARDS). Large SRA^+^ cells were observed only in patients who had died of multiple organ dysfunction syndrome, while small SRA^+^ cells were observed in all healthy control. These findings suggest that large SRA^+^ and TNF-α^+^ cells play important roles in the development of multiple organ injury [15]. The objective of this study was to examine systemic organs and peripheral blood collected in patients who have died of acute exacerbation of IPF or CTD-associated ILD, to identify the underlying pathophysiologic mechanisms.

## Methods

### Study patients

Fourteen autopsy cases of acute exacerbation were examined (12 of IPF, and two of CTD-associated ILD). Patients whose immediate causes of death was considered to be severe bronchopneumonia, aspiration pneumonia or lung cancer were excluded from the analysis.

The presence of current infectious was evaluated using several techniques. Sputum microbiology and blood culture were performed in all patients, and bronchoalveolar lavage in 10. No specific pathogens were isolated in sputum, bronchoalveolar lavage fluid or blood. Cytomegalovirus antigenemia and β-D-glucan tests were negative in all patients.

### Methods

#### Autopsy

Autopsy was performed 2–3 h after death. Major organs were removed and sections were examined after staining with hematoxylin and eosin, Gram stain, methenamine silver-nitrate Grocott’s variation stain, and phosphotungstic acid hematoxylin (PTAH) stain.

#### Cytology

We elected to examine peripheral blood specimens taken before and after the diagnosis of acute exacerbation of IPF or CTD-associated ILD, and at autopsy, for the presence of SRA^+^ and TNF-α^+^ cells. Stored peripheral blood samples (median: 2 samples before diagnosis of acute exacerbation, range: 1–5 specimens, median: 3 specimens after diagnosis, range: 1–9 specimens), and those obtained from the right atrium at autopsy were collected in tubes containing EDTA (Vacutainer plastic, EDTA 2 K, Becton Dickinson, USA). Papanicolaou-stained smears were prepared: erythrocytes were lysed with lysing reagent (826 mg NH_4_CL + 3.7 mg EDTA-4Na + 100 mg KHCO_3_ in 100 ml H_2_O), nucleated cells were suspended in isotonic sodium chloride solution, and suspensions containing approximately 5 × 10^6^ nucleated cells were smeared on glass slides using Auto smear CF-12 (Sakura Seiki, Tokyo, Japan). Cells that did not adhere to glass slides were gently washed off with 95 % ethanol solution. Smear preparations were fixed in 95 % ethanol solution and stained according to the Papanicolaou method.

#### Immunohistochemical and immuno-cytochemical examination

Papanicolaou-stained smears and paraffin sections were examined using the simple stain MAX-PO method (Nichrei Co., Tokyo, Japan) with diaminobenzidine as the chromogen using mouse monoclonal anti-human glycoprotein 1b (CD42b, a platelet marker, Novo Castra, Newcastle-upon- Tyne, UK, 1:100), mouse monoclonal anti-human keratin (AE1/AE3, an epithelial marker, DAKO USA, Carpinteria, CA, USA, 1:150), mouse monoclonal anti-human transmembrane glycoprotein (CD34, an endothelial cell marker, DAKO USA, Carpinteria, CA, USA), mouse monoclonal anti-human TNF-α (Abcam, Cambridge, UK, 1:100) and mouse monoclonal anti-human SRA (CD204, a macrophage SRA marker, 1:200, Trans Genic Inc., Kumamoto, Japan) antibodies. An antigen retrieval method using citrate buffer and microwave heating was employed for all antibodies. As a negative control, the primary antibody was substituted with phosphate- buffered saline, and a positive stain was not observed in these controls.

#### Diagnoses

The diagnoses of IPF and its acute exacerbation were made according to the statements of national and international respiratory medicine societies [6, 7]. The diagnosis of acute exacerbation of CTD-associated ILD was made using the same criteria as IPF. We examined the number of necrotic cardiac myocytes in 1 cm2 PTAH stained sections. When necrotic myocytes were observed as a small group and the exact number could not be counted, we described them as “many,” as shown in Table 1. SRA^+^ cells in peripheral blood that were over twice as large as monocytes were defined as large SRA^+^ cells. We defined alveolar tissue as injury as an absence of alveolar epithelial cells, with peri-alveolar cytokeratin-positive cell debris and mild edema and extravasation. We defined capillary injury as a reduction in number or absence of tube-like or ring-like structures. Alveolar and capillary injuries were identified in immunohistochemical preparations stained with AE1/AE3 or CD34 antibodies. The diagnosis of multiple organ injury was made if 2 or more organs were injured.

**Table 1:** Histopathologic and cytologic findings

	Age	Sex	AE-D	Lung	Heart	Liver	Kid.	Sto.	Large SRA^+^ cells		TNF-α^+^ cells	
									Before AE	At Death	After AE	At Death
1	83	M	4	DAD(E)	<1	−	−	−	ND	5	ND	1
2	82	M	26	DAD(E,P)	many	+	+	−	1	66	0	4
3	83	M	35	DAD(E,P)	2	−	−	−	ND	12	ND	2
4	63	M	24	DAD(E,P)	many	+	+	−	2	609	1	5
5	58	F	7	DAD(E,P)	many	+	+	+	0	2	0	3
6	45	M	30	DAD(E,P)	7	+	+	+	1	44	4	2
7	59	F	27	DAD(E,P)	many	+	−	+	4	1015	1	7
8	73	M	20	DAD(E,P)	many	−	−	−	2	84	2	3
9	64	M	60	DAD(E,P)	47	+	+	+	1	413	3	2
10	68	M	16	DAD(E,P)	38	+	+	−	1	91	0	4
11	61	M	30	DAD(E,P)	20	+	+	−	1	189	2	5
12	63	F	50	DAD(E,P)	8	−	−	−	5	546	5	8
13	74	M	75	DAD(E,P)	31	−	+	−	3	72	2	1
14	87	M	7	DAD(E)	many	+	+	−	5	4	1	2

AE-D represents the duration from the diagnosis of acute exacerbation to death (days). *Abbreviations*: *AE* acute exacerbation, *Kid* Kidney, *Sto* Stomach, *SRA* Scavenger receptor A, *TNF* Tumor necrosis factor, *M* Male, *F* Female, *DAD* Diffuse alveolar damage, *E* Exudative phase, *P* Proliferative phase, *ND* No data

## Results

### Patient characteristics

Three patients presented with acute respiratory symptoms that were diagnosed as an acute exacerbation of IPF; they had not previously been diagnosed with a respiratory disease. The other 11 patients developed acute exacerbation during management of previously-diagnosed IPF or CTD-associated ILD. Median period from the diagnosis of IPF to acute exacerbation of the 11 patients was 14 month (range: 2–26 months). Two patients were acute exacerbation of CTD-associated ILD. All patients had received corticosteroid therapy before the exacerbation, and high-dose corticosteroids and cyclophosphamide after diagnosis of acute exacerbation. All had been treated with oxygen for 1–12 days before death. After reviewing the pathologic findings and clinical records, we determined that chemotherapy and oxygen had not substantially influenced the pathologic findings in any case.

### Pathologic findings in the lungs

The usual interstitial pneumonia pattern and the exudative phase of DAD were observed in all cases; the proliferative phase of DAD was observed in 12 cases (85.7 %, Table 1).

**Fig. 1:**
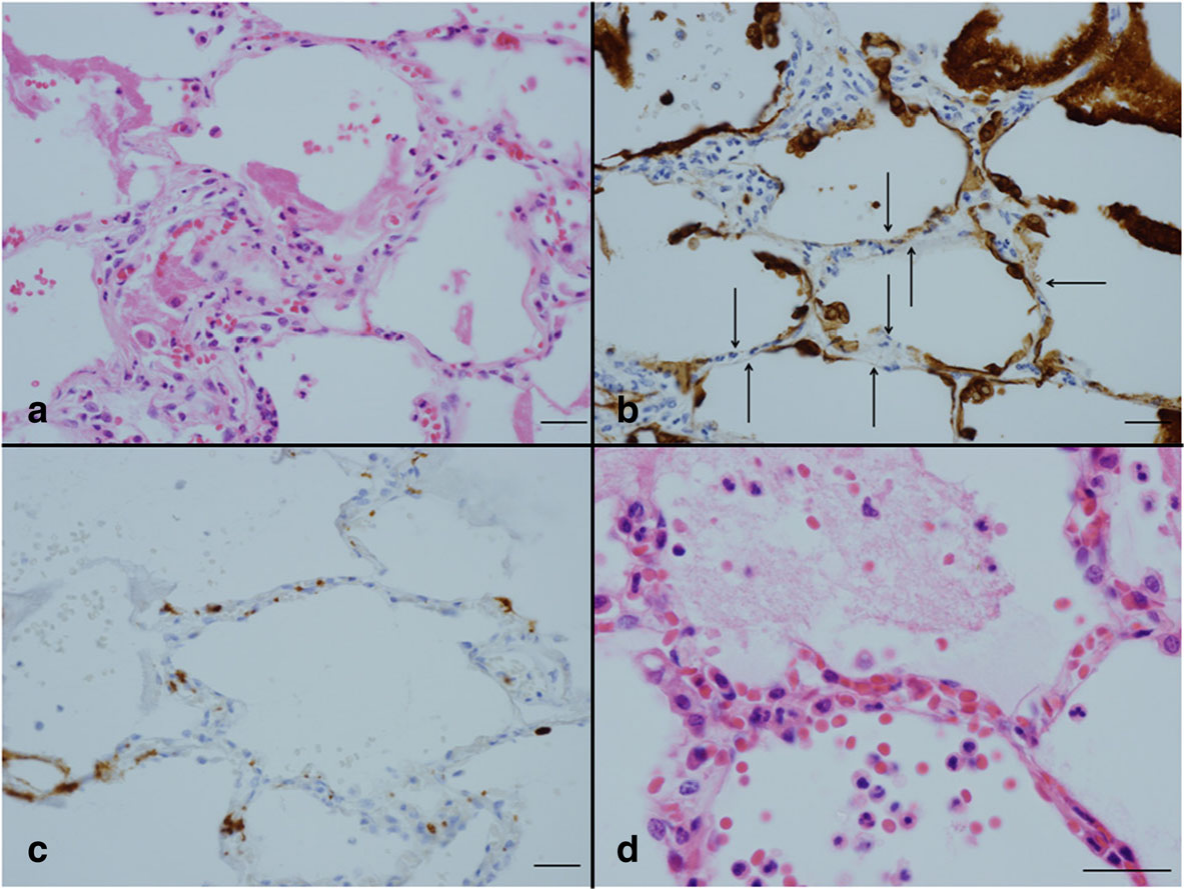
Acute inflammatory lung injury of types 1 (**a**-**c**) and type 2 (**d**). **a** The exudative phase of diffuse alveolar damage with a hyaline membrane with neutrophils accumulation in alveolar septa and air spaces. **b** Alveolar epithelial cells absent from alveoli (*arrow*) and cytokeratin positive cell debris in air spaces. **c** Accumulation of platelets mostly in the capillaries of the alveolar septa. **d** Mild congestion, edema and extravasation with neutrophils accumulation in alveolar septa and air spaces. *Scale bars*: 30 μm (**a**-**d**). Immunohistochemistry for AE1/AE3 (**b**) and CD42b (**c**)

**Fig. 2:**
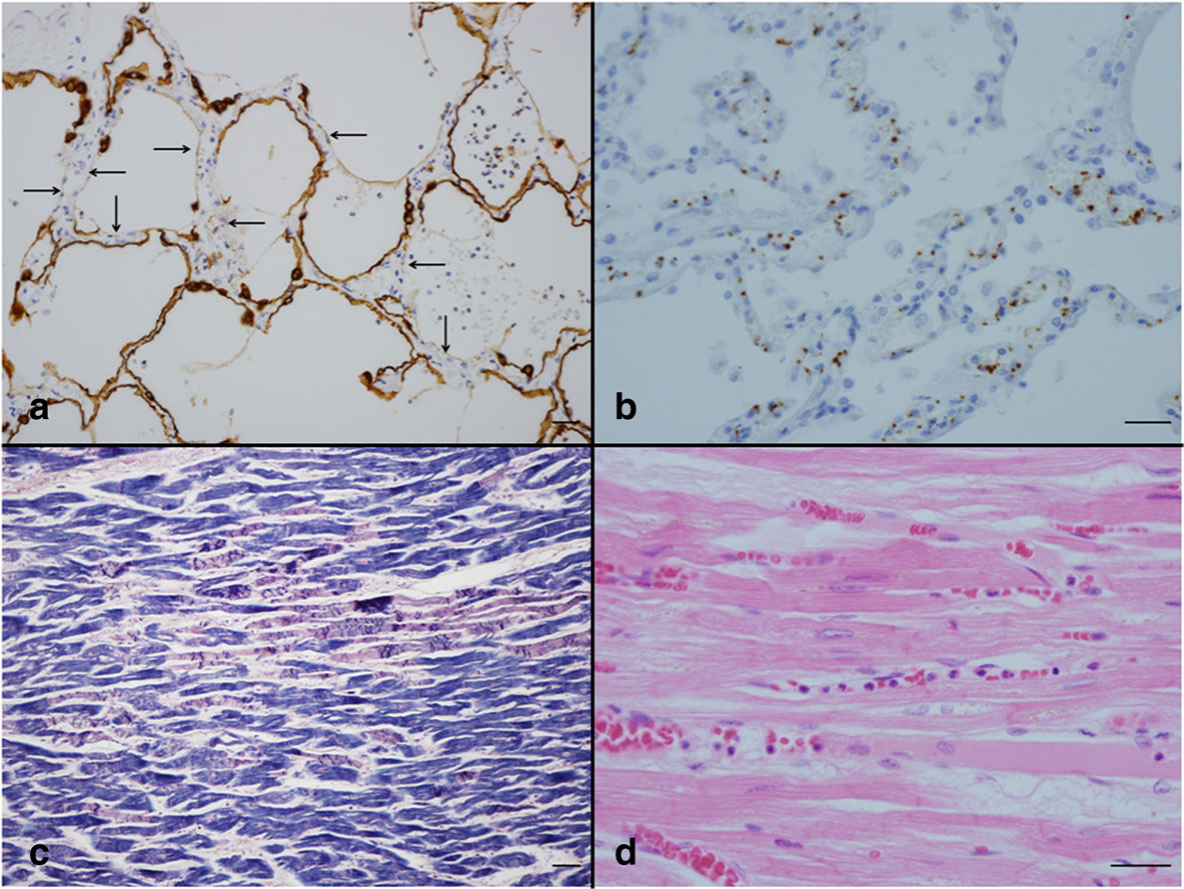
Type 2 acute inflammatory lung injury (**a**, **b**) and cardiac injury (**c**, **d**). **a** Alveolar epithelial cells absent from alveoli (*arrow*) and cytokeratin positive cell debris in air spaces. **b** Accumulation of platelets mostly in the capillaries of the alveolar septa. **c** Contraction band necrosis of a variety of durations. **d** Accumulation of neutrophils mostly in capillaries. *Scale bar*: 30 μm (**a**-**d**). Immunohistochemistry for AE1/AE3 (**a**) and CD42b (**b**). Phosphotungstic acid hematoxylin (**c**)

Acute inflammatory lung injury was classified into one of two types. The first exhibited pathologic findings consistent with the exudative stage of DAD, such as hyaline membranes, congestion, varying amounts of extravasation and fibrin deposition, intra-alveolar edema, neutrophils in the alveolar septal walls and air spaces (Fig. 1a), the injury and disappearance of alveolar epithelial cells (Fig. 1b), and platelets accumulation predominantly in the capillaries of the alveolar septal walls (Fig. 1c). In the second type, all of the above mentioned findings apart from hyaline membranes were observed (Figs. 1d, 2a, and b).

### Pathologic findings in the heart

**Fig. 3:**
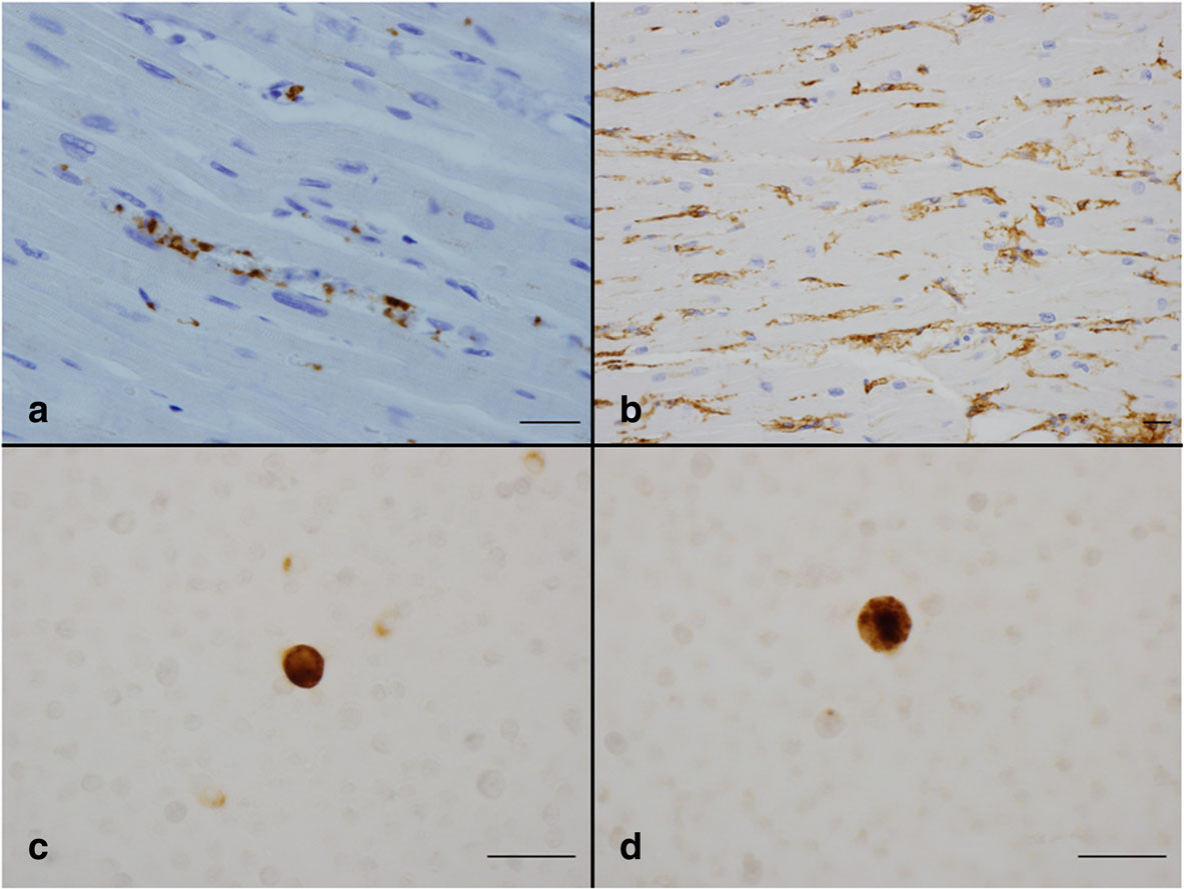
Findings in the heart (**a**, **b**) and peripheral blood (**c**, **d**). **a** Accumulation of platelets mostly in capillaries. **b** Marked reduction of ring-like or tube-like capillary structures, injured capillary endothelial cells have a ragged cytoplasm. **c** A large scavenger receptor-A positive cell. **d** A tumor necrosis factor-α-positive cell. *Scal bars*: 30 μm. Immunohistochemistry for CD42b (**a**), CD34 (**b**), scavenger receptor A (**c**), and tumor necrosis factor-α (**d**)

Contraction band necrosis of varying duration was observed in both ventricular walls (Fig. 2c). Congestion and accumulation of neutrophils (Fig. 2d) and platelets (Fig. 3a) in capillaries were observed in all cases. Numerous tube-like or ring-like structures were observed among cardiac myocytes in normal cardiac tissue; however these structures were markedly reduced in number, and injured capillary endothelial cells had ragged cytoplasm (Fig. 3b).

### Pathologic findings on other organs

Necrosis of hepatocytes was observed in nine cases (64.3 %), necrosis of renal tubular epithelial cells was observed in nine cases (64.3 %) and a shallow gastric ulcer was detected in four cases (28.6 %, Table 1). Accumulation of neutrophils and platelets in capillaries or sinusoidal capillaries were observed in injured organs. There was no evidence of severe tissue injury or sepsis in any case. Specific pathogens were not observed in any organs.

### Cytological findings of PB

About 1.0 × 10^6^ nucleated cells were smeared in one 1.2 × 1.2 cm^2^ area. Only one to three large SRA^+^ cells and TNF-α^+^ cells were observed in some of the smears prepared from peripheral blood specimens taken before death. Large SRA^+^ cells were observed in 11 cases before acute exacerbation was diagnosed (78.6 %, Fig. 3c). TNF-α^+^ cells were detected after the diagnosis of acute exacerbation in nine patients (64.3 %, Fig. 3d). Large SRA^+^ cells and TNF-α^+^ cells were detected in all cases at autopsy (Table 1).

## Discussion

Although CTD-associated ILD and IPF are considered to have different underlying pathogenesis, acute exacerbation has been reported in patients with CTD-associated ILD and other ILDs [3–5]. We judge that the histopathologic findings that we detected were consequence of acute exacerbation, since the histopathological findings of type 1 and 2 acute inflammatory lung injury were different from those of diagnostic histologic findings of idiopathic pulmonary fibrosis (usual interstitial pneumonia) [16]. We were unable to detect any histopathologic or cytologic differences between the 12 cases of IPF and two cases of CTD-associated ILD in our cohort. We propose that the same pathophysiologic mechanism underpin acute exacerbation in these diseases.

We classified acute inflammatory lung injury into two types, which had similar histopathologic and cytologic features except for the presence of hyaline membranes. Consequently, we judge that the same basic cellular and pathophysiologic mechanisms underpin these two types of injury. The earliest changes of DAD identifiable in the lung are reportedly detectable by electron microscopy; at the ultrastructural level, there was evidence of injury and necrosis of type I and II pneumocytes [17, 18]. Hyaline membranes form 2–3 days after injury. Therefore, we propose that the second type of lung injury that we observed is the earliest stage of DAD.

We found DAD and contraction band necrosis in all cases, necrosis of hepatocytes and renal tubular epithelial cells and shallow gastric ulcers in a substantial proportion. Cytokine abnormality were considered to play central role in these tissue or cell injury since accumulation of neutrophils and platelets in capillaries or sinusoidal capillaries were observed in injured organs. It is thought that IPF is a specific form of interstitial pneumonia limited to the lung [7]. Although our cohort was small, our findings suggest that acute exacerbation is a systemic disorder that causes multiple organ injury.

We detected SRA^+^ cells and TNF-α^+^ cells in the systemic circulation in all cases. Neutrophils and platelets had accumulated mainly in capillaries in the alveolar septa and among cardiac myocytes. Capillary endothelial cells were injured. Contraction band necrosis is associated with reperfusion injury [19]. It is widely believed that pro-inflammatory cytokines released by macrophages cause neutrophils to adhere to capillaries and become activated. Activated neutrophils release oxidants and proteases that contribute to tissue damage [20, 21]. We propose that the pathophysiologic mechanisms underlying multiple organ injury in our cases begin with a rapid and substantial release of cytokines into the systemic circulation by activated macrophages causing an imbalance between pro- and anti-inflammatory mediators. Neutrophils and capillary endothelial cells would be activated by these pro-inflammatory cytokines; the adhesion of neutrophils to capillary endothelial would cause them to accumulate in the capillary beds of many organs. Subsequent release of oxidants and proteases by adherent activated neutrophils damages endothelial cells, and then interstitial and parenchymal cells, with platelets also accumulating in capillaries. The consequent tissue injury likely results in multiple organ injury including DAD, and reperfusion of the cardiac micro-circulation also likely results in contraction band necrosis. The numbers of large SRA^+^ cell at autopsy were different with each other, but we could not find any correlation between the abundance of large SRA^+^ cells and degree of organ damage.

Our histopathologic and cytologic findings in acute exacerbation of IPF and CTD-associated ILD are similar to those of MODS [15], suggesting that pathophysiologic mechanisms may be shared to some extent. A variety of primary insults may provoke MODS [20]. We speculated that the SRA^+^ cells and TNF-α^+^ cells that we detected in the systemic circulation had differentiated from monocytes stimulated by the primary insult, and there was an apparent primary insult in each of our MODS cases [15]. Nonetheless, despite careful examination, we were unable to find any evidence of extra- pulmonary primary insults that could have driven the differentiation of SRA^+^ and TNF-α^+^ cells from monocytes in the systemic circulation in our present cases with acute exacerbation.

Scavenger receptor A is the marker of macrophage [22]. Macrophage differentiate from monocyte by the stimulation of macrophage colony stimulating factor (M-CSF) [23]. SRA^−^monocytes became positive for SRA after 5 days in culture with M-CSF [24], and differentiated into large macrophages by 10 days [25]. So we believe that large SRA^+^ cells in systemic circulation indicate the presence of lasted high M-CSF level. In our opinion it will be important to clarify the mechanism underlying the differentiation and activation of SRA^+^ cells and TNF-α^+^ cells in the systemic circulation to illuminate the pathogenesis of acute exacerbation of IPF and CTD-associated ILD.

## Conclusion

Acute exacerbation of IPF and CTD-associated ILD appears to be a systemic disorder that brings about multiple organ injury, with SRA^+^ cells and TNF-α^+^ cells in the systemic circulation playing central roles. Our findings illuminate the importance of these cells in the pathophysiology of acute exacerbation.

## Abbreviations

ARDS: Acute respiratory distress syndrome
CTD: Connective tissue disease
DAD: Diffuse alveolar damage
ILD: Interstitial lung disease
IPF: Idiopathic pulmonary fibrosis
M-CSF: Macrophage colony stimulating factor
MODS: Multiple organ dysfunction syndrome
SRA: Scavenger receptor A
TNF: Tumor necrosis factor

## References

[1] Kim DS, Collard HR, King TE (2006). Classification and natural history of the idiopathic interstitial pneumonias. Proc Am Thorac Soc.

[2] Kondoh Y, Taniguchi H, Kawabata Y, Yokoi T, Suzuki K, Takagi K (1993). Acute exacerbation in idiopathic pulmonary fibrosis. Analysis of clinical and pathologic findings in three cases. Chest.

[3] Suda T, Kaida Y, Nakamura Y, Enomoto N, Fujisawa T, Imokawa S (2009). Acute exacerbation of interstitial pneumonia associated with collagen vascular diseases. Respir Med.

[4] Park IN, Kim DS, Shim TS, Lim CM, Lee SD, Koh Y (2007). Acute exacerbation of interstitial pneumonia other than idiopathic pulmonary fibrosis. Chest.

[5] Churg A, Müller NL, Silva CI, Wright JL (2007). Acute exacerbation (acute lung injury of unknown cause) in UIP and other forms of fibrotic interstitial pneumonias. Am J Surg Pathol.

[6] Collard HR, Moore BB, Flaherty KR, Brown KK, Kaner RJ, King TE (2007). Acute exacerbation of idiopathic pulmonary fibrosis. Am J Crit Care Med.

[7] Raghu G, Collard HR, Egan JJ, Martinez FJ, Behr J, Brown KK (2011). An official ATS/ERS/JRS/ALAT statement: idiopathic pulmonary fibrosis: evidenced-based Guidelines for diagnosis and management. Am J Respir Crit Care Med.

[8] Ambrosini V, Cancellien A, Chilosi M, Zompatori M, Trisolini R, Saragoni L (2003). Acute exacerbation of idiopathic pulmonary fibrosis: report of a series. Eur Respir J.

[9] Rice AJ, Wells AU, Bouros D, du Bois RM, Hansell DM, Polychronopoulos V (2003). Terminal diffuse alveolar damage in relation to interstitial pneumonias. Am J Clin Pathol.

[10] Ashbaugh DG, Bigelow DB, Petty TL, Levine BE (1967). Acute respiratory distress in adults. Lancet.

[11] Sloane PJ, Gee MH, Gottlieb JE, Albertine KH, Peters SP, Burns JR (1992). A multicenter registry of patients with acute respiratory distress syndrome: physiology and outcome. Am Rev Respir Dis.

[12] Montgomery AB, Stager MA, Carrico CJ, Hudson LD (1985). Causes of mortality in patients with the adult respiratory distress syndrome. Am Rev Resir Dis.

[13] Matthay MA, Zimmerman GA, Esmon C, Bhattacharya J, Coller B, Doerschuk CM (2003). Future research directions in acute lung injury. Summery of a National Heart, Lung and Blood Institute working group. Am J Respir Crit Care Med.

[14] Oda K, Ishimoto H, Yamada S, Kushima H, Ishii H, Imanaga T (2014). Autopsy analysis in acute exacerbation of idiopathic pulmonary fibrosis. Respir Res.

[15] Emura I, Usuda H (2010). Histopathological and cytological examination of autopsy cases with multiple organ dysfunction syndromes. Pathol Int.

[16] Katzenstein A-LA. Idiopathic interstitial pneumonia. In: Katzenstein A-LA, editor. Non-neoplastic lung disease. 4th ed. Saunders; 2006. p. 51–84

[17] Tomashefski JF (2000). Pulmonary pathology of acute respiratory distress syndrome. Clin Chest Med.

[18] Schnells G, Voigt WH, Redl H, Schlag G, Glatzel A (1980). Electron microscopic investigation of lung biopsies in patients with posttraumatic respiratory insufficiency. Acta Chir Scand.

[19] Schoen FJ, Mitchell RN, Kumar V, Abbas A, Fausto N, Aster JC (2010). The heart. Pathologic basis of disease.

[20] Husain AN, Kumar V, Abbas A, Fausto N, Aster JC (2010). The lung. Pathologic basis of disease.

[21] Ware LB, Matthay MA (2000). The acute respiratory distress syndrome. N Engl J Med.

[22] Kunjathoor VV, Febbraio M, Poderz EA (2002). Scavenger receptor A-I/II and CD36 are the principal receptors responsible for the uptake of modified low density lipoprotein leading to lipid loading in macrophages. J Biol Chem.

[23] Gordon S, Perry VH, Rabinowitz S, Chung LP, Rosen H (1988). Plasma membrane receptors of the mononuclear phagocyte system. J Cell Sci Suppl.

[24] Naito M, Kodama T, Matsumoto A, Doi T, Takahashi K (1991). Tissue distribution, intracellular localization, and in Vitro expression of Bovine macrophage scavenger receptor. Am J Pathol.

[25] Young DA, Lowe LD, Clark SC (1990). Comparison of the effects of IL-3, granulocyte-macrophage colony- stimulating factor, and macrophage colony-stimulating factor in supporting monocytes differentiation in culture. J Immunol.

